# Skimmed Milk as a Diet in Disease

**Published:** 1871-04

**Authors:** 


Skimmed Milk as a Diet in Disease.—Dr. S. Weir Mitchell writes that he has good success with this treatment in gastric disorders, diarrhœa, malarious and renal dropsies, and nervous maladies. He uses the milk as free as possible from cream, and prefers to have it stand in a well-chilled refrigerator for twenty-four hours. It is given at any temperature the patient likes, and when nausea is marked, a little lime water is added. The quantity given is at first only one or two tablespoonfuls every two hours; this is increased each day until sixteen ounces are given, each day; then the intervals are lengthened so that this quantity is taken in a less number of doses. After three weeks of absolute milk diet, a thin slice of stale bread each day is allowed; after another week, rice once a-day is added, and, later, a chop, and finally a steak; then a gradual return is made to a diet mostly of milk, which is kept up for some months.

At first the patients lose in weight, but after a few weeks they begin to gain and slowly come back to their original weight The urine may be passed frequently at first, but after a few days, no trouble is experienced from this source. The tongue is usually for a time coated with a white fur, but this disappears in a few weeks. The bowels are almost always constipated during the treatment, and it is sometimes very difficult to secure regular alvine evacuations. The pulse is quickened by the diet until sufficient milk is taken each day to prevent any loss in weight, when it falls to its normal rapidity. There is, with most patients, for a few days, an intense sleepiness, but this is of no consequence.

There is always an intense craving for other foods, which makes it very difficult to keep patients with little fortitude steadily upon the milk-diet; this is the greatest obstacle to the success of the treatment; with many patients it is one prolonged struggle, even after some other food is allowed.

He has found more beneficial effects from this treatment in stomachal disorders than any other. A case is related of a lad whose stomach became so irritated and irritable that he could retain no food, and he was vomiting away. On the milk-diet he rapidly recovered. Another is told of a woman, whose dyspepsia, frequent vomiting, and almost constant suffering, was fast wearing her out. Alkalies, tonics, stimulants, acids, pepsin, bismuth, and arsenic were used in vain. She improved rapidly under the milk-diet; but her disgust for it was unconquerable, and constipation was so obstinate that it had to be given up. One case is related of frequent attacks of inflammation in the ileo-cœcal region of the intestine, with sallow skin, which was relieved by this treatment.—*Medical Times.*

Arthur Scott Duncan, M.D., also records in the *Richmond and Louisville Medical Journal* several cases of diabetes mellitus, Bright's disease, disease of the supro-renal capsules, fatty degeneration, etc., successfully treated by the milk-diet.

"In one case of Bright's disease the patient had become enormously œdematous and ascetic; the urine was scanty, highly albuminous, and had a specific gravity of 1010. There were found abundant fatty and hyaline tube-casts. The patient was suffering from cephalagia, nasia, and his countenance was pale, pasty and puffy.

Five pints of skimmed milk was ordered to be taken in divided doses each day; all other articles of diet were prohibited. A diuretic was likewise ordered, composed of twenty grains acetate of potash and twenty minims tincture of digitalis, to be taken three times a-day. This course was pursued for a fortnight, when all traces of albumen in the urine had disappeared, and there was not a trace of dropsy left; neither did any again return. The daily quantity of urine, which had been, before commencing the treatment, five pints, was now reduced to three pints, but its density had increased to 1015.

At this point the diuretic was left off, the milk contined, and a moderate quantity of brown bread allowed at each meal. A tonic of sulphate of quinine and sulphate of iron was ordered. In a month he was dismissed, cured. In six months there had been no reappearance of the disease.

In a second case, the patient's condition was very much like the first, only perhaps worse. Exactly the same treatment was prescribed as with the former patient. The improvement was prompt and positive.

But on eating clandestinely of starchy food far beyond the amount allowed, he had a relapse, which brought back his disease with increased severity. When the exclusive milk-diet was again resorted to, the patient went on to complete recovery, to all appearances.

The first effect of skimmed milk, taken to the extent of five or seven pints daily, is to cause a powerful diuretic effect; the effect of the profusion of water is to flush the uriniferous tubules, and wash out the material that blocks them up. This relieves the pressure on the secondary capillaries, more and more blood flows through them, and the congestion of the capillaries within the malpighian tufts is removed, and less and less albumen transudes through their walls. While this beneficial change is going on, healthy epithelium is developed in the tubules which secrete the urinary excrement from the blood.

Milk is superior to other diuretics, because while it removes a large amount of water from the blood, it renews the supply of albumen in this fluid by the amount of this element which is contained in its substance, restoring a healthy condition of the blood, and preventing a return of the dropsy.

